# Effects of Upper-Extremity Rehabilitation Using Smart Glove in Patients With Subacute Stroke: Results of a Prematurely Terminated Multicenter Randomized Controlled Trial

**DOI:** 10.3389/fneur.2020.580393

**Published:** 2020-11-09

**Authors:** Min-Gu Kang, Seo Jung Yun, Sang Yoon Lee, Byung-Mo Oh, Hyun Haeng Lee, Shi-Uk Lee, Han Gil Seo

**Affiliations:** ^1^Department of Rehabilitation Medicine, Seoul National University College of Medicine, Seoul National University Hospital, Seoul, South Korea; ^2^Department of Rehabilitation Medicine, Seoul National University College of Medicine, Seoul Metropolitan Government Seoul National University Boramae Medical Center, Seoul, South Korea; ^3^Department of Rehabilitation Medicine, Konkuk University Medical Center, Seoul, South Korea

**Keywords:** rehabilitation, stroke, occupational therapy, upper extremity, subacute care

## Abstract

**Background:** Although there have been many trials and interventions for reducing upper-extremity impairment in stroke survivors, it remains a challenge. A novel intervention is needed to provide high-repetition task-specific training early after stroke.

**Objective:** This study aimed to investigate the effect of smart glove training (SGT) for upper-extremity rehabilitation in patients with subacute stroke.

**Methods:** A prospective, multicenter, randomized, controlled study was conducted in patients with upper-extremity hemiparesis with Brunnstrom stage for arm 2–5 in the subacute phase after stroke. Eligible participants were randomly allocated to the SGT group or the control group. The SGT group underwent 30 min of standard occupational therapy plus 30 min of upper-extremity training with smart glove. The control group underwent standard occupational therapy for 30 min plus upper-extremity self-training (homework tasks at bedside) for 30 min. All participants underwent each intervention 5 days/week for 2 consecutive weeks. They were evaluated before, immediately after, and 4 weeks after the intervention. The primary outcome measure was the change in the score of the Fugl-Meyer assessment of the upper extremity (FMA-UE).

**Results:** Twenty-three patients were enrolled. Repeated-measures analysis of covariance after controlling for age and disease duration showed significant time × group interaction effects in the FMA-UE, FMA-distal, and FMA-coordination/speed (*p* = 0.018, *p* = 0.002, *p* = 0.006). Repeated-measures analysis of variance showed significant time × group interaction effects in the FMA-UE, FMA-distal, and Box and Block Test (*p* = 0.034, *p* = 0.010, *p* = 0.046). Mann-Whitney *U*-test showed a statistically higher increase in the FMA-UE and FMA-distal in the SGT group than in the control group (*p* = 0.023, *p* = 0.032).

**Conclusion:** Upper-extremity rehabilitation with a smart glove may reduce upper-extremity impairment in patients with subacute stroke.

**Clinical Trial Registration**: ClinicalTrials.gov (NCT02592759).

## Introduction

Stroke is one of the leading causes of death and disabilities worldwide (1). Upper-extremity dysfunction is a common complication after stroke (2, 3). The incidence of upper-extremity dysfunction has been reported to be up to 80% in stroke survivors (4). This leads to disability and reduced quality of life because upper-extremity function is crucial for activities of daily living (ADLs) (5). Therefore, restoring upper-extremity function is an important goal of stroke rehabilitation.

Conventional occupational therapy has been a primary treatment to improve upper-limb function in stroke survivors. However, the method and quality of treatment differ depending on the therapist or clinic, and the treatment is also labor-intensive (6). As the prevalence of stroke increases, occupational therapists are increasingly burdened with the growing demand for occupational therapy for stroke survivors (7). Moreover, it is difficult to provide sufficient repetition or intensity of conventional occupational therapy to produce functional improvement (8). Therefore, there is an increasing need for a novel intervention that is effective and standardized but is less labor-intensive.

A variety of interventions for upper-extremity rehabilitation have been introduced to overcome the limitations of conventional occupational therapy for promoting the recovery of arm and hand function after stroke (9). In particular, constraint-induced movement therapy and task-specific training programs have shown evidence for enhancing upper-limb motor recovery. Consequently, highly repetitive task-specific training is required to minimize impairment (10, 11). However, it is not easy to provide sufficient high-repetition task-specific training for all patients. In addition, despite various rehabilitation efforts, about one-half of stroke survivors show no recovery of upper-limb function at 6 months after stroke (12).

Robot-assisted training using robotic devices enables highly repetitive, intensive, and task-specific training with less labor-intensive (13, 14). Hand exoskeletons have been introduced in response to the expectations for improving dexterity and ADLs. Traditional hand exoskeletons have mechanisms of rigid linkage-based or wire driven (15, 16). Rigid components and rigid linkages are used in those mechanisms. Due to the rigidity and heavyweight, the devices impede natural hand movement and ADLs. In addition, the large size interfered with visual feedback and prevented them from comfortable wearing.

The smart glove used in the present study is a soft glove with bending sensors for monitoring individual finger movements and built-in inertial measurement unit sensors for capturing wrist and hand motions. It can provide intensive and repetitive training through the patients' own efforts without the assistance of therapists (8, 17). Additionally, it can measure the range of motion, thus enabling the quantitative evaluation of motor recovery. Besides, it allows active training with visual feedback while the patients are playing the game content. Adaptive level control by an artificial intelligence component in the software provides appropriate training tailored to the patient's condition. As a result, patients are provided individualized repetitive task-specific training that has been known to enhance neuroplasticity while they are enjoying the game.

In this study, we aimed to investigate the effect of smart glove training (SGT) for upper-extremity rehabilitation in patients with subacute stroke by comparing this training method with homework tasks.

## Materials and Methods

This study was a prospective, multicenter, single-blind, randomized controlled trial conducted between October 2015 and June 2018 at 2 university hospitals in Korea. The study protocol was registered at ClinicalTrials.gov (NCT02592759) and approved by the institutional review board of each hospital (approval nos. J-1507-002-684 and 16-2015-74/071) in accordance with good clinical practices and the Helsinki Declaration. Written informed consent was obtained from every participant or legal representative.

### Participants

Patients who were hospitalized for stroke from October 2015 to June 2018 were recruited from the two centers. The inclusion criteria were (1) age ≥19 years, (2) unilateral hemiparesis caused by a first-ever stroke (ischemic, hemorrhagic) that was confirmed on computed tomography or magnetic resonance imaging, (3) in the subacute phase after 72 h and within 3 months from stroke onset, (4) upper-extremity hemiparesis with Brunnstrom stage for arm 2–5, and (5) can tolerate sitting for at least 1 h to receive treatment. The exclusion criteria were (1) inability to perform tasks during occupational therapy because of severe hemineglect or hemianopia, (2) upper-extremity contracture due to severe limitation of motion, (3) spasticity in the wrist and fingers with Modified Ashworth Scale score > 2, (4) Fugl-Meyer assessment (FMA)-wrist and hand score ≥ 21, (5) moderate to severe cognitive dysfunction with Mini-mental State Examination score <18, (6) severe aphasia, and (7) a diagnosis of a malignant tumor.

### Randomization

Eligible participants were randomly allocated to either the SGT group or control group with a block randomization size of 4. Permuted block randomization is useful to ensure the balance of the number of patients assigned to each group (18). By selecting a block size of 4, every 2 participants in one block would be assigned to the intervention and control groups in random order. In this manner, the desired allocation to each group is guaranteed. An independent researcher who was not in contact with any patient performed the randomized allocation. The ratio between the SGT and control groups was 1:1 at each hospital. The principal investigator, outcome assessors, and data analysts were blinded to the group allocations of the participants until statistical analysis.

### Intervention

The participants in the SGT group underwent 30 min of conventional occupational therapy plus 30 min of upper-extremity training with the smart glove, whereas those in the control group underwent 30 min of conventional occupational therapy plus 30 min of upper-extremity rehabilitation homework (self-training after receiving instructions from an occupational therapist). Each intervention was conducted for 5 days/week for 2 consecutive weeks. Conventional occupational therapy such as stacking cone, graded range of motion arc, or pegboard activities was provided by occupational therapists according to the ability of the participant.

**Figure 1:**
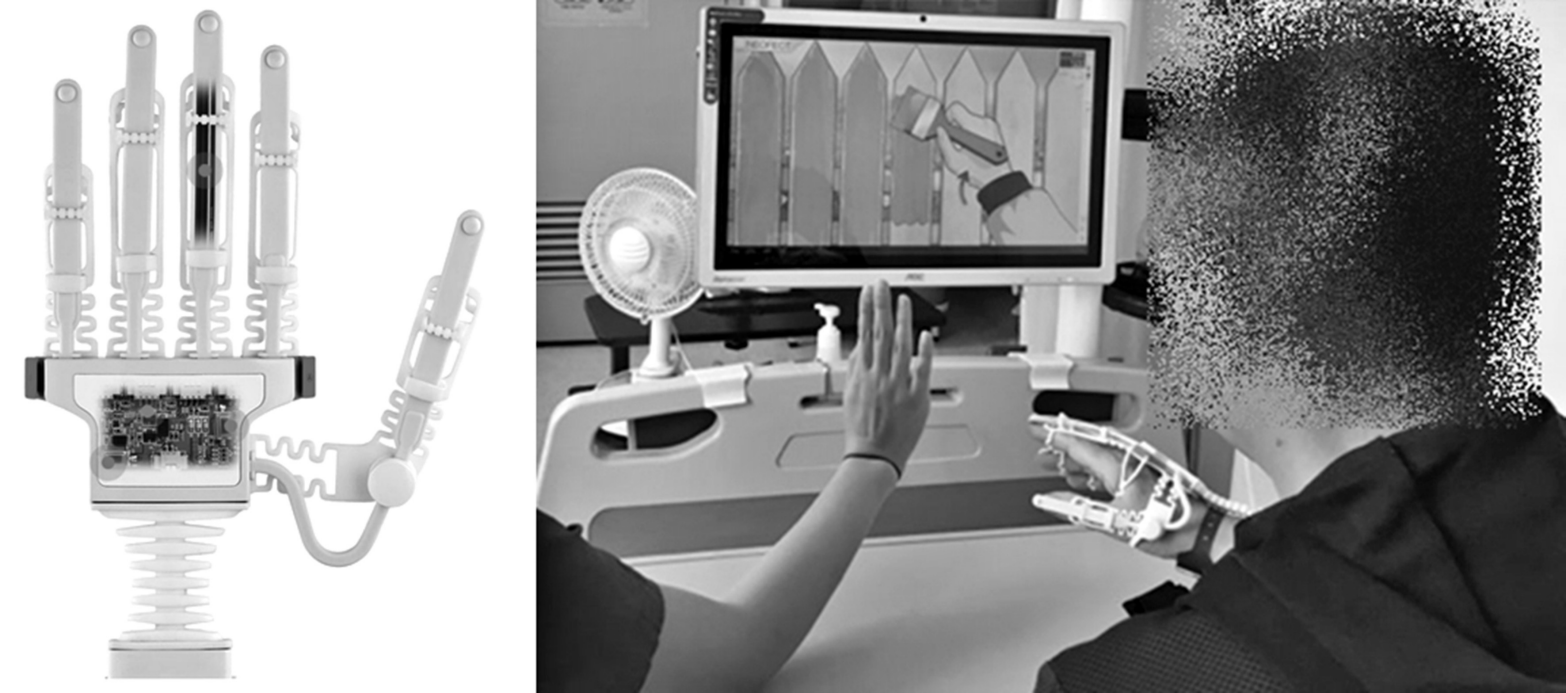
The RAPAEL smart glove and a patient in smart glove training.

The smart glove (RAPAEL™; Neofect, Seongnam, Rep. of Korea) was used in the experimental intervention group. It monitors the movements of the fingers, hand, and wrist. The glove has flexible bending sensors in the finger parts, which are variable resistors that change with bending and computes the amount of individual finger movements. The wrist part of the smart glove has inertial measurement unit sensors that detect 9-axis movement and the position of the hand and wrist. Data from the sensors of the smart glove are transferred *via* Bluetooth to the application installed in a tablet personal computer. Thereafter, motion analysis is conducted including measurement of active and passive range of motion. With these bio-mechanical evaluations, the application provides visual feedback by showing hand and wrist movements of a patient in real time on a monitor while the patient is conducting various motion tasks related to ADLs (Figure 1). The representative motion tasks include forearm supination and pronation, wrist flexion and extension, wrist radial and ulnar deviation, and finger flexion and extension.

The participants in the control group conducted rehabilitation homework tasks using the affected hand. The homework tasks consisted of following 10 items: (1) grasping and releasing a grip ball, (2) wiping a table using a soft towel, (3) pushing a rubber clay, (4) putting large beads into a cup, (5) imitating spooning up, (6) imitating drinking water from a cup, (7) putting pins in diamond-shaped holes of a pegboard, (8) making small dumplings with rubber clay, (9) flipping and matching cards, and (10) turning a notebook 1 sheet at a time. An occupational therapist chose three items according to the ability of the participant. The clinical research coordinator confirmed that self-training was implemented appropriately.

### Outcome Measures

The primary outcome measure was the change in the score of the Fugl-Meyer assessment of the upper extremity (FMA-UE). The FMA-UE is the most frequently used assessment tool for motor impairment after hemiplegic stroke (19, 20). It has shown excellent inter-rater reliability and validity in patients with stroke (21, 22). Thirty-three items are rated on a 3-point ordinal scale (0 = cannot perform, 1 = performs partially, 2 = performs fully). The FMA-UE (score, 0–66) was subdivided into FMA-proximal (shoulder, elbow, and forearm; score, 0–36), FMA-distal (wrist and hand; score, 0–24), and FMA-coordination/speed (score, 0–6). Higher scores indicate better motor function.

The secondary outcome measures included the changes in the scores of the FMA-proximal, FMA-distal, FMA-coordination/speed, Jebsen-Taylor Hand Function Test, Box and Block Test, grip strength, Modified Barthel Index-upper extremity (MBI-UE), and Carer Burden Scale. The FMA-proximal, distal, and coordination/speed subscales were analyzed as secondary measures to determine which subdomains were changed. The Jebsen-Taylor Hand Function Test provided a measure of hand function required for ADLs (23). It is a reliable and valid tool in patients with hemiparesis after stroke (24). The time taken to perform seven tasks was measured. A scoring system that ranges from 0 to 105 (each subset score, 0–5) was used in this trial (25). The Box and Block Test was used to measure gross manual dexterity. It has been shown to be reliable and valid in patients with stroke (26). The number of 1-inch blocks transported from 1 box to the adjacent box within 60 s was measured (27). Grip strength was used to evaluate arm function after stroke (28). Maximum grip strength is reliable in hemiparetic patients with stroke (29). The strength (lb) of the affected hand was measured using a dynamometer. The MBI provided a measure of the ability to perform ADLs (30). It has shown excellent inter-rater reliability and concurrent validity in subjects after stroke (31, 32). The maximum total score of MBI-UE ranged from 0 to 30, and the maximum subscale score was 5 (personal hygiene and bathing) or 10 (dressing and feeding). The Carer Burden Scale was used to measure the burden of care among the caregivers (33). It consisted of 4 items (cleaning the palm, cutting fingernails, dressing, and cleaning under the armpit), and each item was graded from 0 (no care burden) to 4 (maximum care burden). The total score of Carer Burden Scale ranges from 0 to 16, and higher scores indicate a higher feeling of burden.

All outcome measures were evaluated before (T1), immediately after (T2), and 4 weeks after (T3) the intervention.

### Sample-Size Calculation

A previous study reported that additional upper-extremity rehabilitation with an ergonomic glove resulted in an additional increase of 6.7 points in the FMA (8). We conducted a sample-size estimation to achieve 80% power with a 2-tailed α of 0.05, by using the result of an ergonomic glove that was similar to the smart glove used in the present study. Considering a 20% dropout rate, the sample size was estimated to be 24 participants in each group, for a total of 48 participants.

### Statistical Analysis

Baseline characteristics were compared between the SGT and control groups by using Pearson's chi-square test for categorical variables and the Mann-Whitney *U*-test for continuous and ordinal variables. The changes in outcome measures among time points were compared using repeated-measures analysis of variance (RM-ANOVA) and repeated-measures analysis of covariance (RM-ANCOVA) in the intention-to-treat populations. The last observation carried forward method was used to impute missing values. Statistical significance was accepted at *p* < 0.05. All statistical analyses were performed using the Statistical Package for the Social Sciences version 20.0 (IBM Corp, Armonk, NY, USA).

## Results

**Figure 2:**
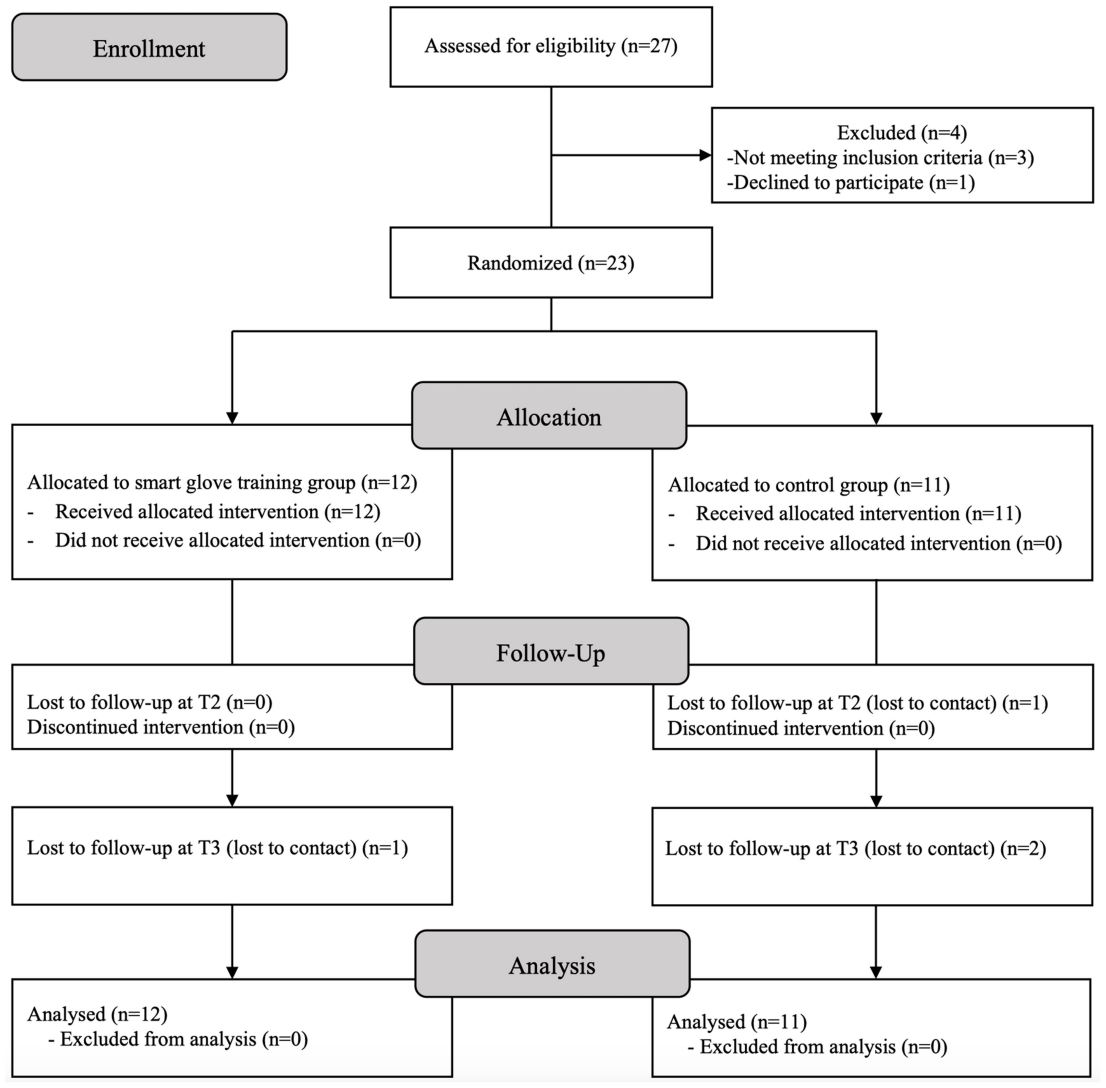
CONSORT (Consolidated Standards of Reporting Trials) flow diagram.

The trial was prematurely terminated owing to slow recruitment. A total of 23 participants were finally included in the study, and all participants completed the entire training sessions. One participant was lost to follow-up at T2, and three participants were lost at T3. Statistical analysis was performed for the 23 participants according to intention-to-treat analysis (Figure 2).

Table 1 shows the baseline characteristics of the participants in each group. Despite random allocation, differences were observed in age and disease duration. Participants in the SGT group were significantly younger than those in the control group (50.92 ± 16.68 vs. 64.64 ± 13.83 years; *p* = 0.044), whereas the disease duration of the SGT group was longer than that of the control group (30.75 ± 20.01 vs. 19.00 ± 9.85 days; *p* = 0.059). To offset the possible selection bias, RM-ANCOVA with age and disease duration as confounding variables was performed in the final analysis.

**Table 1:** Baseline characteristics of the participants (*N* = 23).

**Characteristics**	**SGT group (*n* = 12)**	**Control group (*n* = 11)**	***p*-Value**
Age (years)	50.92 ± 16.68	64.64 ± 13.83	0.044^*^
Sex			0.537
Male	7 (58.3%)	5 (45.5%)	
Female	5 (41.7%)	6 (54.5%)	
Hemiplegic side			0.469
Right	5 (41.7%)	3 (27.3%)	
Left	7 (58.3%)	8 (72.7%)	
Stroke type			0.827
Hemorrhagic	6 (50.0%)	5 (45.5%)	
Infarct	6 (50.0%)	6 (54.5%)	
Disease duration (days)	30.75 ± 20.01	19.00 ± 9.85	0.059
MMSE	24.83 ± 3.33	26.27 ± 3.17	0.260

*Variables are presented as number (%) or mean ± standard deviation*.

*SGT, smart glove training; MMSE, Mini-mental State Examination*.

* *p < 0.05*.

**Figure 3:**
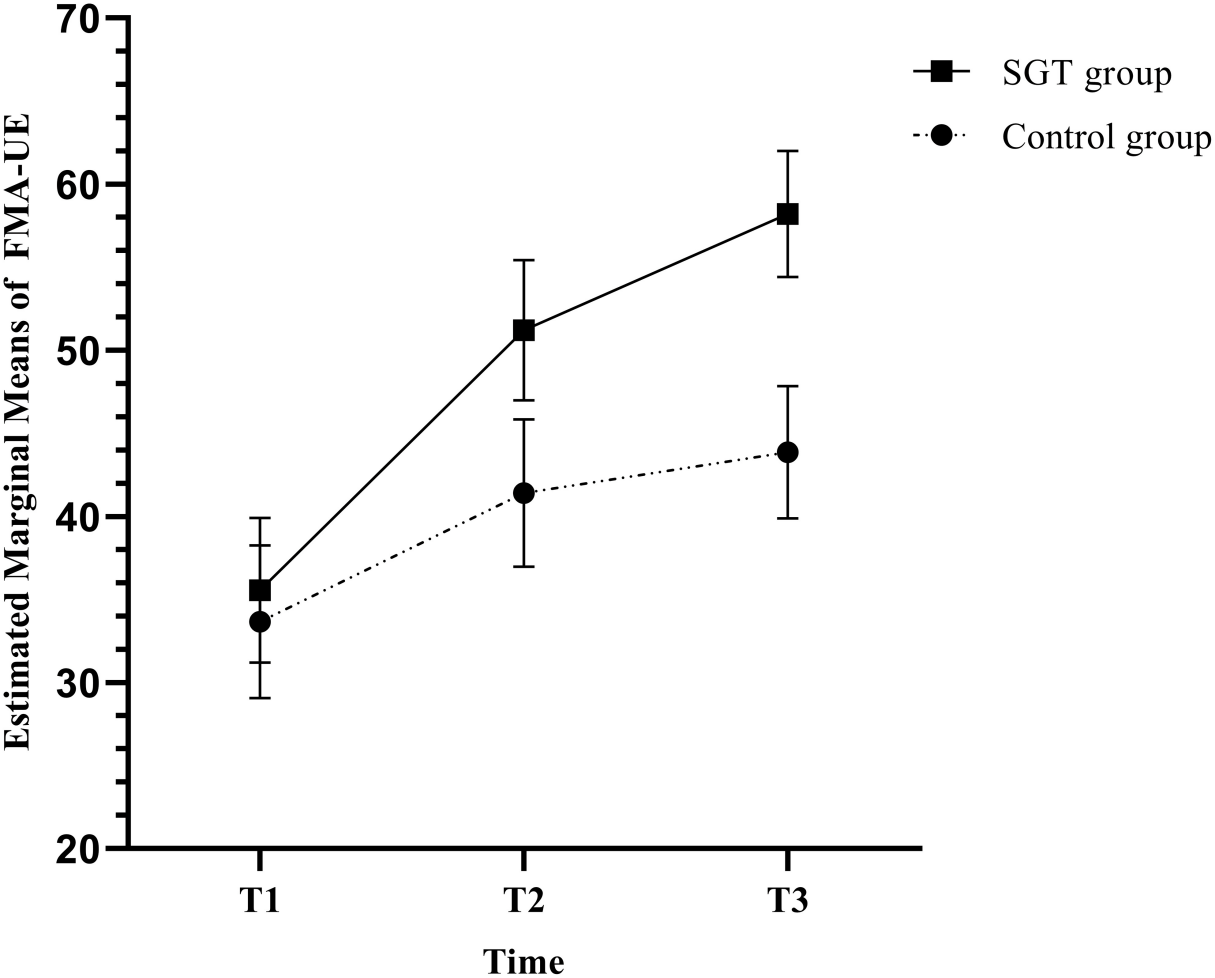
Estimated marginal means and standard errors of the Fugl-Meyer assessment of the upper extremity (FMA-UE) in repeated-measures analysis of covariance (RM-ANCOVA) with correction for age and disease duration.

Table 2 shows the outcome measures at each time point and the results of the Mann-Whitney *U*-test, RM-ANOVA, and RM-ANCOVA. In RM-ANCOVA after controlling for age and disease duration, the FMA-UE, which was the primary outcome measure, showed a significant time × group interaction effect (*F* = 4.479, *p* = 0.018). RM-ANOVA also showed a significant interaction effect of group and time in FMA-UE (*F* = 3.653, *p* = 0.034). The Mann-Whitney *U*-test showed a statistically higher increase of the FMA-UE score in the SGT group than in the control group at T3 (*p* = 0.023) but not at T2 (*p* = 0.316). Figure 3 shows the estimated marginal means of the FMA-UE after controlling for age and disease duration over time.

**Table 2:** Changes in outcome measures across time points in the SGT and control groups.

**Time**	**SGT group (*n* = 12)**	**Control group (*n* = 11)**	**Contrasts^**a**^**	***P***	**Unadjusted *F*^**b**^**	***P***	**Adjusted *F*^**c**^**	***P***
FMA-UE					3.653	0.034^*^	4.479	0.018^*^
T1	33.83 ± 13.99	35.55 ± 15.06						
T2	47.83 ± 14.26	45.09 ± 15.40	T1 to T2	0.316				
T3	55.42 ± 11.20	46.91 ± 14.98	T1 to T3	0.023^*^				
FMA-proximal					0.441	0.580	0.703	0.465
T1	23.58 ± 8.01	21.73 ± 7.50						
T2	29.25 ± 7.34	27.64 ± 7.30	T1 to T2	0.928				
T3	31.00 ± 6.54	27.73 ± 6.68	T1 to T3	0.608				
FMA-distal					5.182	0.010^*^	7.169	0.002^*^
T1	8.50 ± 6.60	11.09 ± 7.50						
T2	15.33 ± 7.06	15.73 ± 6.99	T1 to T2	0.211				
T3	19.17 ± 6.25	16.09 ± 7.40	T1 to T3	0.032^*^				
FMA-coordination/speed					2.973	0.062	5.780	0.006^*^
T1	1.75 ± 2.01	2.73 ± 2.05						
T2	3.25 ± 2.22	3.64 ± 1.86	T1 to T2	0.347				
T3	3.92 ± 1.68	3.09 ± 2.34	T1 to T3	0.079				
Jebsen Hand Function Test					1.329	0.271	1.641	0.213
T1	7.00 ± 11.70	9.09 ± 17.48						
T2	25.92 ± 26.95	26.91 ± 27.97	T1 to T2	0.928				
T3	40.08 ± 30.02	29.64 ± 33.10	T1 to T3	0.288				
Box and Block Test					3.560	0.046^*^	2.917	0.072
T1	10.75 ± 12.93	9.91 ± 15.20						
T2	19.08 ± 17.14	22.91 ± 19.43	T1 to T2	0.235				
T3	31.33 ± 19.19	23.82 ± 15.87	T1 to T3	0.260				
Grip strength					0.645	0.530	0.803	0.455
T1	12.33 ± 10.49	14.71 ± 18.04						
T2	19.75 ± 13.62	23.80 ± 25.35	T1 to T2	0.695				
T3	24.67 ± 14.54	24.03 ± 20.88	T1 to T3	0.608				
MBI-UE					2.165	0.138	1.546	0.229
T1	16.83 ± 6.24	11.55 ± 5.66						
T2	20.75 ± 5.59	20.00 ± 6.07	T1 to T2	0.051				
T3	25.17 ± 5.95	21.64 ± 4.84	T1 to T3	0.786				
Carer Burden Scale					0.537	0.588	0.813	0.451
T1	10.50 ± 3.87	12.82 ± 4.33						
T2	7.25 ± 3.60	9.91 ± 4.32	T1 to T2	0.695				
T3	6.17 ± 2.95	9.36 ± 4.72	T1 to T3	0.566				

*Variables are presented as mean ± standard deviation*.

*SGT, smart glove training; FMA-UE, Fugl-Meyer assessment-upper extremity; MBI-UE, Modified Barthel Index-upper extremity*.

a *Comparisons of the changes between groups with the Mann-Whitney U-test*.

b *Time × group interaction in repeated-measures analysis of variance*.

c *Time × group interaction adjusted for age and disease duration in repeated-measures analysis of covariance*.

* *p < 0.05*.

In RM-ANCOVA after controlling for age and disease duration, the FMA-distal and FMA-coordination/speed showed significant time × group interaction effects (*F* = 7.169, *p* = 0.002; *F* = 5.780, *p* = 0.006). RM-ANOVA showed a significant time × group interaction effect in the FMA-distal (*F* = 5.182, *p* = 0.010) but not in the FMA-coordination/speed (*F* = 2.973, *p* = 0.062). The Mann-Whitney *U*-test showed a statistically higher increase of the FMA-distal score in the SGT group at T3 (*p* = 0.032) but not at T2 (*p* = 0.211). The FMA-distal showed similar statistical results with the FMA-UE, but the FMA-proximal and FMA-coordination/speed did not. RM-ANCOVA showed no significant interaction effects in the other secondary outcome measures including the FMA-proximal (*F* = 0.703, *p* = 0.465), Jebsen Hand Function Test (*F* = 1.641, *p* = 0.213), Box and Block Test (*F* = 2.917, *p* = 0.072), grip strength (*F* = 0.803, *p* = 0.455), MBI-UE (*F* = 1.546, *p* = 0.229), and Carer Burden Scale (*F* = 0.813, *p* = 0.451). Adverse events or serious adverse events did not occur in all participants during the trial.

## Discussion

The results of this study showed that SGT produced greater improvements of upper-extremity impairment, according to the FMA-UE, FMA-distal, and FMA-coordination/speed, than control tasks in patients with subacute stroke within 3 months from onset. The improvements in the FMA-UE and FMA-distal were significantly greater in the SGT group than in the control group at 4 weeks after the intervention. However, greater improvements were not observed immediately after the intervention. Our hypothesis for this result is that better but not statistically greater improvements in motor impairment immediately after SGT might have encouraged the participants to consistently use their paretic arm and hand, which gradually widened the gap of recovery between the SGT and control groups. On the other hand, the number of participants might be insufficient to prove the significance of the difference at immediately after the intervention because of the early termination of the study.

A previous trial showed that SGT was superior to conventional occupational therapy in improving upper-extremity function and quality of life in patients with chronic stroke (17). Although upper-extremity function measured using the Box and Block Test showed a marginally significant difference between the 2 groups, the analysis did not reveal greater improvement of ADLs. The findings of the present study are in concordance with those of a previous study in which ADLs did not show statistically greater improvements after SGT than after control tasks. To guarantee better recovery of ADLs, greater improvement of proximal-arm function might be needed, which was not a primary goal of SGT. In addition, further ADL training may be necessary to translate the improvement of upper-extremity impairment to improvement of ADLs.

The timing and dose of rehabilitation are important factors in gaining functional recovery after stroke. Starting rehabilitation early after stroke is important for functional recovery (34). Earlier rehabilitation is correlated with better-preserved cortical maps, and the training effect of rehabilitation decreases over time (35). The dose and intensity of arm training is also a critical factor to optimize rehabilitation efficacy (36). Animal studies suggested that a critical threshold of rehabilitation intensity was required for poststroke recovery and a high dose of arm training leads to effective recovery of arm function and neuroplastic changes (37–39). It is recommended that patients on an inpatient stroke rehabilitation meet the standard of 1 h of occupational therapy per day but generally they receive less than the required time (40). A review article reported that stroke survivors participate in upper-extremity training during occupational therapy <11 min in the acute phase and 12 min in the subacute phase (41). Besides, there is a substantial amount of inactive time outside of occupational therapy time. In this study, one of the explanations for the effect of SGT may be that the smart glove training had compensated for the lack of required dose and intensity of rehabilitation.

The possible mechanism of greater improvement in SGT may be based on motor learning principles. Feedback and practice are known to be important for motor learning in occupational therapy (42, 43). Intrinsic feedback includes visual information and sensory information from muscles, joints, and tendons. During SGT, intrinsic feedback is scarcely disturbed owing to the small size, lightweight, and elasticity of the device. Extrinsic feedback enhances the intrinsic feedback through external sources such as directions from therapists or biofeedback from devices. Visual feedback via the display screen during SGT helps in correcting the movements as an extrinsic feedback (44). Skill is known to improve in relation to the amount of practice (45) and repetitive massed practice is required to enhance brain reorganization (38, 46). In this trial, SGT enabled intensive massed practice through correcting the motions from intrinsic and extrinsic feedback, and this effect might be extended to promote improvement of motor impairments.

This study has several limitations. First, the sample size was not sufficient to validate the effect of SGT. It was difficult to recruit eligible participants because of the narrow inclusion/exclusion criteria. In addition, most stroke patients with mild to moderate impairment were discharged from the tertiary university hospitals before study enrollment. Therefore, the trial was prematurely terminated before reaching the initially estimated sample size of 48 patients. Although the results of this study showed the significant effect of SGT on the primary outcome measure, the lack of significance in the secondary outcome measures might have resulted from insufficient statistical power. Second, the baseline patient age was statistically different between the SGT and control groups. Age and disease duration are critical for recovery, especially in the subacute period after stroke. Therefore, RM-ANCOVA was performed to rule out the effect of age and disease duration. Third, the interventions in both groups included conventional occupational therapy, which precluded direct comparison between SGT and homework tasks. In the strict sense, this study compared the additional effect of SGT or homework tasks on conventional occupational therapy. This was ethically unavoidable because there is no evidence of the effect of SGT alone for improving upper-extremity function. Fourth, SGT trains only the distal part of the upper extremity. Combining proximal function training may be more efficient in improving upper-extremity function and ADLs in subacute stroke patients. A further study combining SGT and proximal arm training is expected to optimize upper-extremity rehabilitation.

## Conclusion

This study suggests that SGT may be a safe and effective intervention for upper-extremity rehabilitation, especially for the improvement of distal motor impairment in patients with subacute stroke. Recovery of distal arm and hand function rather than proximal arm function may be the therapeutic target. Larger clinical trials are needed to confirm the effect of SGT based on this study.

## Data Availability Statement

The raw data supporting the conclusions of this article will be made available by the authors, without undue reservation.

## Ethics Statement

The studies involving human participants were reviewed and approved by (1) The institutional review board of Seoul National University Hospital (2) The institutional review board of Seoul National University Boramae Medical Center. The patients/participants provided their written informed consent to participate in this study.

## Author Contributions

M-GK analyzed data and drafted the manuscript. SY recruited patients and collected data. SL and B-MO designed the study protocol and supervised execution of the study. HL recruited patients and collected data. HS supervised execution of the study, interpreted data, and revised the manuscript. S-UL supervise design of the study and interpreted data. All authors read and approved the manuscript.

## Conflict of Interest

The authors declare that the research was conducted in the absence of any commercial or financial relationships that could be construed as a potential conflict of interest.
